# When Age Meets Atrocity: Medical Realities for Israel’s Older Hostages

**DOI:** 10.5041/RMMJ.10559

**Published:** 2025-10-31

**Authors:** John Adekoya

**Affiliations:** MB1 Doctor, Ipswich Hospital, Ipswich, United Kingdom

**Keywords:** Antisemitism, elder abuse, medical harm

**Dear Editor**,

I read “Kidnapped But Not Kids: A Case Series of Three Octogenarian Hostages Held in Captivity by Hamas” by Clarfield and Levine^1^ with what I can only describe as a mix of disbelief and deep concern. It may well represent one of the starkest instances of elder abuse in recent years, if not modern memory. There’s something particularly troubling, even haunting, about the additional layers of trauma these elderly individuals endured, given both their age and medical vulnerability. The sense of disregard for even the most basic ethical standards is difficult to ignore.

We tend to talk a lot about medical ethics in abstract terms, perhaps too often. But incidents like these bring the conversation uncomfortably close to home, reminding us how crucial it is to recognize, call out, and, in some way, try to remedy such wrongs when we come across them, whether in practice or the wider world. While elder abuse is, regrettably, a global issue, as Alias et al. have pointed out, similar unmerited mistreatment is increasingly seen in other sectors of society. For example, young Jewish doctors have faced parallel instances of abusive discrimination within hospital settings.^3^ Sometimes it’s subtle, sometimes not.

I noticed the authors did not hold back in criticizing organizations like the International Committee of the Red Cross and the World Health Organization. To be honest, their frustration feels justified. The muted, almost perfunctory, response from these groups and the public really makes you wonder whether certain populations are simply overlooked or their suffering seen as less newsworthy. Speaking from personal experience here in the UK, I find it striking, maybe disappointing, how little coverage or real discussion these stories seem to get. One has to ask: Are we, as a profession, looking away?

Yet what strikes me most isn’t just the injustice. It’s the sheer resilience shown by those elderly survivors. It’s humbling, really, something that reminds us all why we went into medicine in the first place. Of course, their survival raises difficult questions about justice and how or even if societies properly remember such suffering. Santayana’s line, “Those who cannot remember the past are condemned to repeat it,”^4^ feels awfully pertinent, although I sometimes wonder how often we heed the warning.

Looking ahead, I want to believe that, given the diversity and complexity in today’s healthcare systems, the least we can do as medical professionals is not shy away from addressing these difficult truths. Speaking up and intervening even when it’s uncomfortable may be one small step toward tackling antisemitism and all forms of discrimination that continue to worm their way into our institutions. Only then can we say we’re upholding the spirit of trust and professionalism our field is meant to stand for.
